# Evaluation of an integrated care service facility for people living with hepatitis C in New Zealand

**DOI:** 10.5334/ijic.819

**Published:** 2012-12-11

**Authors:** Robyn Horwitz, Loren Brener, Carla Treloar

**Affiliations:** National Centre in HIV Social Research, University of New South Wales, Sydney, NSW 2052, Australia; National Centre in HIV Social Research, University of New South Wales, Sydney, NSW 2052, Australia; National Centre in HIV Social Research, University of New South Wales, Sydney, NSW 2052, Australia

**Keywords:** hepatitis C, knowledge, lifestyle changes, treatment, integrated care

## Abstract

**Introduction:**

People living with hepatitis C are a highly marginalised population who may not readily access health care. Existing models of hepatitis C care may not meet the needs of these patients. This research evaluates the experiences of patients attending an innovative hepatitis C clinic that offers integrated care and service delivery.

**Method:**

Surveys were completed by 120 clients and comprised of questions relating to changes in lifestyle habits since attending the clinic, hepatitis C knowledge, hepatitis C treatment and experiences with health care staff at the clinic.

**Results:**

The majority of respondents indicated that attendance at the clinic has provided them with the information to better manage their hepatitis C and had given them confidence to make lifestyle changes. Participants demonstrated a very high knowledge of hepatitis C and reported experiencing a less discriminatory environment at the clinic compared to other health care settings. Respondents who had been attending the clinic for more than 6 months were significantly more likely to indicate a desire to commence hepatitis C treatment over the next 5 years.

**Discussion:**

The findings point to the importance of integrated care in the community setting in providing clients with a positive experience of health care, which appears to increase their skills and desire to better manage their hepatitis C.

## Introduction

In Australia and New Zealand approximately 1% of the population is currently living with chronic hepatitis C [1, 2]. In New Zealand, it is estimated that close to 50,000 people are currently living with hepatitis C with 25 new infections per week [3, 4]. Hepatitis C in New Zealand, as elsewhere in the developed world, is primarily attributable to injecting drug use. While there has been a noticeable decline in seroprevalence of hepatitis C among injecting drug users using needle exchange programs to reported levels of approximately 50% between 2004 and 2009 [4] published sources still report approximately 84% of people who inject drugs in New Zealand being infected with hepatitis C [5, 6]. Treatment uptake remains relatively low [7–9]. In Australia 3760 individuals underwent hepatitis C treatment in 2010 although 221,000 people are estimated to be living with chronic hepatitis C [10]. Limited research exists on treatment uptake in New Zealand. However, <10% of methadone maintenance clients in New Zealand have been reported as having received treatment for hepatitis C [6].

The low rate of treatment uptake is likely a combination of the health care system, provider and patient characteristics [11]. Hepatitis C is an illness that attracts a large amount of stigma and discrimination because of its association with injecting drug use [12–15]. Stigma and discriminatory practices within the health sector may have a major impact on access to health care, can affect treatment and health outcomes and can act as a barrier to treatment [16–20]. More so, it has been ascertained that some physicians are not comfortable providing hepatitis C treatment to people who inject drugs [21–23]. Reports from qualitative research suggest that there are many instances where people living with hepatitis C receive minimal or inaccurate information from their doctor on diagnosis, no pre-post test counseling and no referrals to support services [15, 24–26]. Current health systems therefore may not be meeting the needs of these marginalised populations with hepatitis C. Given that many people who inject drugs have more than one chronic illness or a comorbid mental health issue [27], it is very important for this population in particular to be encouraged to access the health system.

Hepatitis C treatment is lengthy and may have adverse side effects and potential risks. Not all individuals with hepatitis C are psychologically ready nor have life circumstances that are facilitative of hepatitis C treatment [11]. People living with hepatitis C require continued support, referral services and access to information to maintain their health prior to treatment [28]. For example, lifestyle changes, such as exercise therapy, reduced alcohol, restricted diet and weight loss have been shown to be an important adjunct management strategy for people with chronic hepatitis C [29, 30]. In addition, support for people with hepatitis C is an important part of the decision process to commence and complete treatment [31–33]. Therefore, the challenge for health care services is to develop a holistic approach to hepatitis C management that assists with living with hepatitis C, in particular focusing on enabling hepatitis C treatment readiness. However, a double-bind currently exists. Without support, patients may not be adequately prepared to engage with treatment services. However, support is typically offered in treatment services located in tertiary settings, for which the barriers to engagement may be too high for patients with complex and chronic needs.

The need to implement alternative models of care have been recognized and trialed in some countries [34–36], typically involving multi-disciplinary teams and treatment sites that are acceptable to patients who are either not able or unwilling to access conventional medical care [37]. Current research on multidisciplinary health-care settings that provides a continuum of care shows increased numbers of people being referred to specialist hepatitis C care, increased numbers of people commencing treatment and improved access for people living with hepatitis C to mainstream health care [9, 34, 38]. An evolving body of research suggests successful treatment outcomes by increasing general practitioners’ capacity to manage and treat hepatitis C [39], involving peer workers [40, 41] and expanding the role of nurses in the management of chronic hepatitis C [42].

This paper contributes to a growing literature exploring innovative models of care for people living with a highly stigmatized condition. Few innovations in this area have established new and separate services, but have built on existing access points for people living with hepatitis C, notably drug and alcohol treatment services. This paper presents an evaluation of the patient experience of an integrated clinic for hepatitis C in Christchurch, New Zealand. The clinic opened in October 2008 as a three-year pilot. During the data collection phase, it was situated in Christchurch opposite a major needle exchange program. The clinic was forced to relocate after the premises were destroyed by earthquakes in February 2011. The initial location of the clinic meant that clients of the Needle Exchange Program need only cross the road to access the clinic and peers from the Needle Exchange Program were able to accompany them.

The clinic is the only community-based model in Christchurch, New Zealand and was set up to provide integration across primary, community, hospital and tertiary care services with the intention of improving health outcomes for those with hepatitis C. Prior to establishment, the clinic conducted a process of ‘mapping’ steps in the patient’s journey to identify existing gaps and weakness in traditional models of care and create protocols for health care based on best practice in order to increase access to and uptake of medical treatment. The clinic was developed as a nurse-led managed care network, with a shared care delivery in alliance with primary healthcare services and treatment providers to offer care that improves access to hepatitis C testing, diagnosis and treatment and enhances health outcomes for those living with hepatitis C. It was staffed by a hepatitis C nurse specialist supported by a social worker, and a general practitioner. The clinic provided structured plans of care that supported clinical protocols and guidance for each stage in the management of a patient with the intention of improving continuity of care across disciplines. A multidisciplinary clinical review of each newly enrolled patient and client follow-up actions were carried out on a weekly basis. A review of client care guidelines was carried out by the social worker and nurse on a monthly basis. An integrated care protocol with hospital-based hepatitis C treatment providers and a Memorandum of Understanding with relevant referring services defined the roles and practices between the clinic and other services. In addition, an advisory group provided a forum for consultation and collaboration to meet operational and strategic goals and to promote best practice within the service. This group included hepatitis C clinical providers and those with a vested interest in hepatitis C in the Christchurch community. Client feedback and focus group meetings facilitated client input into service delivery. The clinic was free and open to anyone who believed they had been exposed to hepatitis C or who wished to undertake testing or management of their hepatitis C. From the commencement of the pilot study to the end of the evaluation period, 520 clients attended the clinic.

This paper presents an evaluation of this model of care by assessing whether the hepatitis C community clinic in New Zealand was able to provide support and encourage lifestyle changes to enable people to live with their illness, provide care in a non-judgmental environment, support clients’ knowledge of hepatitis C treatment and transmission, and willingness to commence hepatitis C treatment.

## Method

As one of the aims of the research was to evaluate how people feel about and their experiences at the clinic, those who had been attending for the longest period were chosen to participate. Therefore the first 130 clients who attended the clinic were invited to participate in the survey in November 2010, irrespective of their hepatitis C status. The administration of data collection took place over a four-month period. Surveys were distributed by post with a replied paid envelope attached and took approximately 15–20 min to complete. A reminder letter was sent to those participants who had not returned the surveys after two weeks and then again after another two weeks. Participants were given a small gift (value NZ$20) to thank them for their participation. The study was approved by the Human Research Ethics Committee of the University of New South Wales, Australia and the Health & Disability Ethics Committee, New Zealand.

The survey included questions relating to changes in lifestyle habits since attending the clinic, hepatitis C knowledge, treatment intention and current treatment status, referral to specialists, experiences with health care staff at the clinic and with health care staff in mainstream care, access pathways to the clinic, hepatitis C testing, and demographic information.

Three questions assessing client satisfaction with the clinic were scaled to form a satisfaction scale with scores ranging from 1 ‘very dissatisfied’ to 5 ‘very satisfied’ (Cronbach’s α=0.83). Two 5-item scales consisting of identical items were used to compare participants’ perceptions of discrimination by clinic staff with discrimination by general healthcare workers. Items were scored on a 5-point Likert scale ranging from 1 ‘disagree strongly’ to 5 ‘agree strongly’, lower scores indicative of more positive attitudes (Cronbach’s α=0.74 and 0.88, respectively). In order to examine the association between treatment intention and time at the clinic, ‘length of time at the clinic’ was collapsed into two categories (<6 months, >6 months). Participants’ intention to commence treatments was considered as a dichotomous variable (yes, no). Correlation of length of time at the clinic and participants’ intention to commence treatment was calculated using point biserial correlation. Descriptive analysis, including χ^2^ and t-tests were performed using SPSS V18.

## Results

### Sample and hepatitis C status

The target sample was comprised of the first 130 clients attending the Hepatitis C Community Clinic who were asked to complete a self-administered survey. One-hundred and twenty surveys were returned (92% response rate). Demographic characteristics of the samples are reported in Table 1. Approximately one-third of the sample (36%) reported injecting drug use within the last month, of which 39.5% injected daily or more, with morphine (MST M-Eslon Kapanol LA’s) being the most commonly injected drug. Forty-one percent reported currently being in a methadone program.

One-third (33.3%) of the respondents had first heard about and been referred to the clinic from the local Needle Exchange Program (see Table 2). Of the 92% of clients tested for hepatitis C, 72.6% reported being hepatitis C positive (see Table 3). Eighty-eight participants (83%) reported being tested <1 year ago, with 64 respondents (61%) being tested at the clinic (see Table 3). The most commonly cited reasons for attending the clinic for the first time was for testing, to obtain more information about hepatitis C and for hepatitis C treatment information (see Table 4).

### Lifestyle changes

Over 70% of respondents indicated that they had either reduced or cut out alcohol, and had regular hepatitis C check-ups since attending the clinic and nearly half (48%) had made changes to their diet (See Table 5). In addition, most respondents (82.6%) reported that attendance at the clinic had provided them with the information to better manage their hepatitis C and 72.8% felt the clinic had given them confidence to make changes in their lives to better manage their condition.

### Experience and satisfaction with clinic and perceived discrimination

Respondents reported high levels of confidence in the knowledge of clinic staff, with 90% of those who had seen the nurse reporting having ‘a lot of confidence’ in her hepatitis C knowledge. Of the respondents who had seen the nurse, 80% found it ‘very easy’ to obtain an appointment with her and none of the respondents found it ‘very difficult’. In addition of the participants who had seen the doctor and social worker, 98% and 94% reported that they had ‘a lot of confidence’ in the clinic doctor and social worker, respectively. More than half (57%) and almost three-quarters (71%) of the sample found it ‘very easy’ to obtain an appointment with the social worker and doctor, respectively.

The satisfaction scale indicates that the majority of participants were very satisfied with the care, support and information received from the clinic with the mean score of 13.7 (scale: 3–15; IQR 12–15). There was a significant difference between perceptions of discrimination by clinic staff compared with general health workers (t=9.9, df=101, p=0.001). Participants reported a mean score on the perceived discrimination measure of 8.6 for clinic staff (scale: 5–25; IQR 5–10) compared with a mean score of 13.5 for general healthcare workers (IQR 10–17). Additionally, the clinic satisfaction scale was significantly correlated with perceiving the clinic staff to be non-discriminatory (r=-0.425, p<0.01) suggesting that viewing clinic staff as non-judgmental and accepting was linked to greater satisfaction.

### Hepatitis C knowledge, treatment intention and referral

Respondents’ knowledge about hepatitis C was high with correct answers provided by at least 89% of the sample on all items (see Table 6).

Of the participants who had ever been tested for hepatitis C (n=108), 42 participants (62.7%) received a referral from the clinic to see a specialist about treatment for their hepatitis C (see Table 7), of which, 33 participants (78.6%) reported that they had seen a specialist. Injecting drug use in the last month was found to have a significant effect on whether clients at the clinic had ever seen a specialist about their hepatitis C. Of the respondents who reported ever going to see a specialist about their hepatitis C, 72.7% had not injected in the last month compared to 27.3% who had injected in the last month (χ^2^=5.7, df=1, p=0.05).

Within this sub sample (n=108), 31 participants (30.1%) were either currently on a waiting list or having pre-tests in order to begin interferon treatment, with 11 participants (10.6%) currently on treatment and 20 participants (19%) reporting having previously been on treatment. From this subsample, 23 participants (22.3%) reported having tried unsuccessfully to get into treatment in the last two years and over half (55.4%) of the respondents reported intending to go on treatment within the next five years. Those who had attended the clinic for more than 6 months were significantly more likely to report planning for treatment within the next 5 years, than respondents who had attended the clinic for <6 months (67.2% vs. 32.8%, x=6.545, df=1, p=0.01). Eighteen percent of this sub sample had previously been told by a health care worker or a specialist that they were not eligible or not suitable for hepatitis C treatment with the main reason given being either their liver did not have enough damage or because they were injecting drugs. Of the participants who had tried to get treatment for hepatitis C over the last two years and not been able to, concerns over side effects and the length of the waiting list were cited as the main reasons for not having had treatment. Over 90% of the total sample reported current enrolment with a GP and more than two-thirds stated that their GP knew that they were attending the clinic.

## Discussion

For many people living with chronic hepatitis C infection, the more traditional models of care can be intimidating, judgmental, inaccessible or inappropriate for their needs [34] and limit the amount of care and support that can be found outside of tertiary settings [42]. This integrated care clinic for people living with hepatitis C appears successful in providing clients with a positive experience of hepatitis C testing and care, with effective referral pathways to treatment and with the motivation to make positive changes in lifestyle. A quarter of clinic clients chose general health care as one of the main reasons for attending the clinic, with 23% looking for support with their hepatitis C and 37% for hepatitis C testing. This highlights the importance of integrated care with a multi-disciplinary team to cater for diverse range of services that is required by this marginalised population. In addition, given the marginalised nature of this client group, it is likely that some of the 44 (37%) clients that came to the clinic specifically to have a test for hepatitis C, may otherwise have never been tested and may have remained unaware of their hepatitis C status or have been provided inaccurate or incomplete information [24, 26]. The data also illustrates that the clinic is a referral source for the client group, with 63% of all referrals to specialists among this client group coming from the clinic. Based on reports from clients, this clinic was able to provide care that was positively endorsed by clients, and was reported to be significantly lower in perceived discrimination as compared to general health care settings.

Trust in health care providers is a significant issue for marginalized communities, affecting many health outcomes including uptake of referral and advice [43–45]. One-third of clients first heard about the clinic from the local needle exchange program showing the importance of the placement of clinic near the needle exchange program for attracting clients. Situating this clinic within the target community and fostering a relationship with the local needle exchange program, may have contributed to the clients perception of the clinic staff as non-discriminatory. Further, clients were comfortable to report drug use, which is important in ensuring appropriate care [46, 47]. There was no significant difference found between current injecting drug users and those reporting no injecting drug use within the last month with regards to treatment intention, satisfaction scale and experiences with staff at the clinic which supports the findings that the clinic staff are non-judgmental and do not discriminate based on drug use. Clients’ satisfaction with the clinic and their perceptions of non-judgmental care may have positively influenced treatment referral outcomes. In this sample over 60% had seen a specialist, representing double the proportion reported in a study of people who inject drugs recently conducted in NSW, Australia [11]. The impact of integration of care can be seen in visibly reducing health inequalities by providing care for a population that routinely faces stigma and discrimination within the health care sector and would be less likely to get treatment.

A commonly cited barrier to treatment uptake is lack of adequate knowledge of hepatitis C treatment, transmission and symptoms [48]. Participants at the clinic demonstrated a very high knowledge of hepatitis C which is in contrast to other research [49–51]. In a recent study in Australia, hepatitis C knowledge was measured among clients of the Medically Supervised Injecting Centre and four opiate substitution clinics in Sydney Australia. Although not directly comparable, the knowledge scores of participants in the Sydney study were remarkably lower than those reported here [52]. For example, only 17% of the Sydney sample could identify that alcohol can contribute to complications of hepatitis C compared to 97% of the Christchurch sample. The high levels of knowledge found in this sample demonstrated the success of integrated care in providing hepatitis C information in a non-judgmental and supportive environment.

Given the strict regimen of and side effects associated with hepatitis C treatment, not everyone may be ready nor want to go on treatment. Treatment is not the only option nor always the best solution for management of hepatitis C [28, 29]. Hence encouraging clients to be both aware of and to implement lifestyle changes to reduce harms associated with chronic hepatitis C is an important component of integrated care. That almost three quarters of the sample engaged in some lifestyle change and directly associated this with clinic attendance indicates the importance of ongoing care for a population that routinely would not access health care.

It is important to note points of concerns with the study. There are limitations in using a satisfaction scale to making inferences about quality of care as they can be subjective, subject to biases and difficult to interpret [53]. In addition, the response rate in the study was high. While such a response rate is not typical, there are likely to be a number of factors which can account for it, such as the positive relationship clients have with the clinic and clinic staff as well as the cash gift provided on completion of the survey [54].

## Conclusion

Despite difficulties in determining the impact of this integrated care model without baseline data to examine changes over time, client reports on the model of care offered by the clinic indicate that it created a supportive environment to conduct hepatitis C testing, provided knowledge and information to enable lifestyle changes and encouraged specialist referral and treatment uptake. Providing acceptable and effective health care for people experiencing multiple disadvantages and marginalization is challenging. This integrated care model has successfully engaged with people living with a stigmatized condition, many of whom also still practice the illegal and socially maligned practice of injecting drug use. This primary health clinic in a community setting sits within a larger effort to increase treatment uptake among people with hepatitis C. However, few programs have established specifically to meet the needs of people with hepatitis C. Based on these findings, the clinic has achieved significant results in effecting both better self-management and referral to tertiary services.

## Acknowledgements

This study was funded by the Canterbury District Health Board. The authors would like to extend our sympathy to the people of Christchurch for the devastating earthquakes and the terrible aftershocks that continue to devastate their beautiful city. We would like to acknowledge and thank all the study participants, with special thanks to the staff at the Hepatitis C Community Clinic. The National Centre in HIV Social Research is supported by a grant from the Australian Government Department of Health and Ageing.

## Reviewers

**Jacqueline Cumming,** Associate Professor, Director, Health Services Research Centre, School of Government, Victoria University of Wellington, PO Box 600, Wellington, 6035, New Zealand

**Stephen McNally**, PhD, Senior Research Fellow, Australian Research Centre in Sex, Health and Society (ARCSHS), La Trobe University, 215 Franklin Street Melbourne VIC, Australia 30000

**One anonymous reviewer**

## Figures and Tables

**Table 1.** Demographics

	n (%)*
Age	
Mean (SD) (IQR)	44 (10.1) (37–52)
Gender	
Male	67 (56.8)
Identity	
New Zealand European	84 (71.2)
New Zealand Maori	12 (10.2)
Other/more than 1	24 (18.6)
Highest level of education	
Primary or secondary school: left without school certificate	48 (40.7)
School certificate without university entrance	24 (20.3)
School certificate with university entrance	10 (8.5)
Attended or completed university	19 (16.1)
Diploma or trade certificate	17 (14.4)
Main source of income	
Benefits (sickness, invalid & domestic purposes)	76 (66.7)
Full time work	25 (21.9)
Part time/casual	8 (7)
Other	4 (3.5)
Length of attendance at the clinic	
<6 months	29 (24.6)
7 months or more	89 (75.4)

*Valid percent.

**Table 2.** Where did you first hear about the hepatitis C clinic?

	n (%)*
Rodger Wright Centre (local Needle Exchange Program)	39 (33.3)
Hepatitis C Resource Centre**	12 (10.3)
General practitioner	5 (4.3)
Family member	4 (3.4)
Friend	20 (17.1)
Methadone clinic	12 (10.3)
Posters/advertisement	9 (7.7)
Other	16 (13.7)

*Valid percent. **A community-based organisation providing information, education, support and advocacy for those infected or affected by hepatitis C.

**Table 3.** Self reported hepatitis C status and location of most recent test for participant who reported being tested for hepatitis C (n=108)

	n (%) n*
Hepatitis C status	
Hepatitis C positive	77 (72.6)
Hepatitis C negative	9 (8.5)
Cleared spontaneously	9 (8.5)
Cleared through treatment	9 (8.5)
Don’t know	2 (1.9)
Location of last hepatitis C test	
Hepatitis C community clinic	64 (61)
General practitioner	11 (10.5)
Hospital	25 (23.8)
Prison	2 (1.9)
Alcohol and drug service	3 (2.9)

*Valid percent.

**Table 4.** Reasons for attending the clinic for the first time

	n (%) n=119*
For information about hepatitis C	36 (30)
To have a test for hepatitis C	44 (36.7)
For hepatitis C treatment information	38 (31.7)
For support	28 (23.3)
To look after general health	30 (25.2)
Other	8 (6.7)

*Valid percent. More than one option could be selected. Values do not add up to 100%.

**Table 5.** Changes in lifestyle habits since attending the clinic

	Participants answered ‘yes’ n (%)*
Changed your diet	47 (48)
Reduced/cut out alcohol	61 (72.6)
Increased level of exercise	42 (44.2)
Used complimentary/alternative medicines for hepatitis C	18 (19.1)
Had hepatitis C check-ups	74 (71.8)

*Valid percent. More than one option could be selected. Values do not add up to 100%.

**Table 6.** Knowledge of hepatitis C

	Correctly answered n (%)*
People living with hepatitis C can damage their liver when they drink alcohol	114 (97.4)
There is a hepatitis C vaccine that can be used to prevent people from getting infected with the hepatitis C virus	97 (89)
Studies show that 60% of people who inject drugs with ‘used needles’ are infected with hepatitis C	108 (93.9)
People can live many years without knowing that they have been infected with the virus	114 (98.3)
Some treatment for hepatitis C, such as interferon, can cause depression as a side effect in some patients	109 (97.3)
Using ‘new’ (i.e., never used before) needles, syringes and equipment reduces the risk of being infected with hepatitis C	109 (94.8)
Coughing and sneezing can spread hepatitis C	111 (97.4)
Hepatitis C treatments can result in the hepatitis C being completely removed (or cleared from one’s blood)	100 (89.3)
The hepatitis C virus can spread from shared kitchen cups, plates or utensils	103 (90.4)
Once someone’s hepatitis C virus has been completely treated and cleared they cannot get re-infected with hepatitis C	107 (94.7)
People can get infected with hepatitis C from tattoos and body piercing	114 (98.3)
Hepatitis C cannot be transmitted by hugs or handshakes	116 (100)
Some hepatitis C genotypes respond better to treatment than others	108 (96.4)

*Valid percent.

**Table 7.** Sources of referral to a specialist in hepatitis C treatment

Of the participants who reported being tested for hepatitis C	n (%)*
Methadone clinic	13 (24.1)
GP	37 (54.4)
Hepatitis C clinic	42 (62.7)
Other	9 (23.1)
Never received a referral	12 (27.9)

*Valid percent. Questions were not mutually exclusive.
